# Technology and evidence in daily cardiovascular practice

**DOI:** 10.1007/s12471-026-02044-8

**Published:** 2026-04-09

**Authors:** Pim van der Harst

**Affiliations:** Department of Cardiology, University Medical Centre Groningen, Groningen, The Netherlands

This issue contains four original articles from Dutch centres covering quite different clinical topics. However, they all focus on finding better ways to improve our daily clinical practice.

Perdeck et al. show that their in-house developed urine production gauge substantially outperforms manual urine output charting (100% versus 40% hourly completeness) with direct electronic health record integration. Promising findings that warrant further evaluation [1].

Kanon et al. provide real-world data on PFO closure over seven years. Recurrent stroke rates align well with randomised trials, though TIA recurrence was somewhat higher. Controlling cardiovascular risk factors such as hypertension remains essential alongside the procedure [2].

Azzahhafi et al. show that pre-hospital HEART score assessment by paramedics yields only moderate agreement with in-hospital scoring, with a missed MACE rate exceeding the accepted safety threshold. This is not new, it was one of the reasons previously the preHEART score was developed, replacing risk factors (difficult to assess in an emergency setting, pre-hospital) with sex for easier ambulance use [3]. However, better training and possibly high-sensitivity troponin assays can also not be ignored [4].

Finally, Kroesen et al. found that adding a smartphone programme to standard cardiac rehabilitation did not improve physical activity, suggesting more intensive mHealth approaches are needed [5].

## References

[1] Perdeck JJ, Sussenbach GA, Bradshaw EN, et al. Automated monitoring of urine output in hospitalized patients with indwelling urinary catheters: a clinical evaluation on the cardiology ward. Neth Heart J. 2026; 10.1007/s12471-026-02038-6. 41949728 10.1007/s12471-026-02038-6 PMC13090461

[2] Kanon MW, Hoendermis ES, Badings EA, van Wijngaarden J. Stroke and transient ischemic attack recurrence after PFO closure in patients with cryptogenic embolism. Neth Heart J. 2026; 10.1007/s12471-026-02042-w. 41949729 10.1007/s12471-026-02042-w PMC13090459

[3] Sagel D, Vlaar PJ, van Roosmalen R, et al. Prehospital risk stratification in patients with chest pain. Emerg Med J. 2021;38:814–9. 34373266 10.1136/emermed-2020-210212 PMC8551969

[4] Azzahhafi J, Chan Pin Yin DRPP, Epping M, et al. Performance of the HEART score in pre-hospital settings for suspected non-ST elevation acute coronary syndrome: the POPular HEART study. Neth Heart J. 2026; 10.1007/s12471-026-02041-x. 41973194 10.1007/s12471-026-02041-x PMC13090458

[5] Kroesen SH, Vonk T, Nuijten MAH, et al. A cardiac-rehab home-based mHealth program to improve physical activity in patients with coronary artery disease: a randomized controlled trial. Neth Heart J. 2026; 10.1007/s12471-026-02039-5. 41954703 10.1007/s12471-026-02039-5 PMC13090453

